# Dissection of intercostal nerves by means of assisted video thoracoscopy: experimental study

**DOI:** 10.1186/1749-7221-8-3

**Published:** 2013-02-13

**Authors:** Juan Pablo Cáceres, Santos Palazzi, Jose Luis Palazzi, Manuel Llusá, Sanz M, Varci S

**Affiliations:** 1 Centro Médico Teknon, Barcelona, Spain; 2 Faculty of Medicine, Barcelona, Spain; 3 Department Experimental Surgery, Hospital Gregorio Marañón, Madrid, Spain

## Abstract

In total brachial plexus preganglionic lesions (C5-C6-C7-C8 and T1) different extraplexual neurotizations are indicated for partial motor function restitution. Mostly for the flexion of the elbow. Neurotization with intercostal nerves (ICN) to musculocutaneous nerve has been known and accepted during many years with different results 2 - 5. The customary technique as described by various authors is carried out by means of a large submammary incision to harvest three or four intercostal nerves (Figure 1). Then are connected by direct suture or grafts to the musculocutaneous nerve or its motor branches 6 - 7. In this article the authors described the possibility of dissection intercostal nerves by means of assisted video thoracoscopy. (VATS-videdo assisted thoracic surgery).

## Background

**Figure 1:**
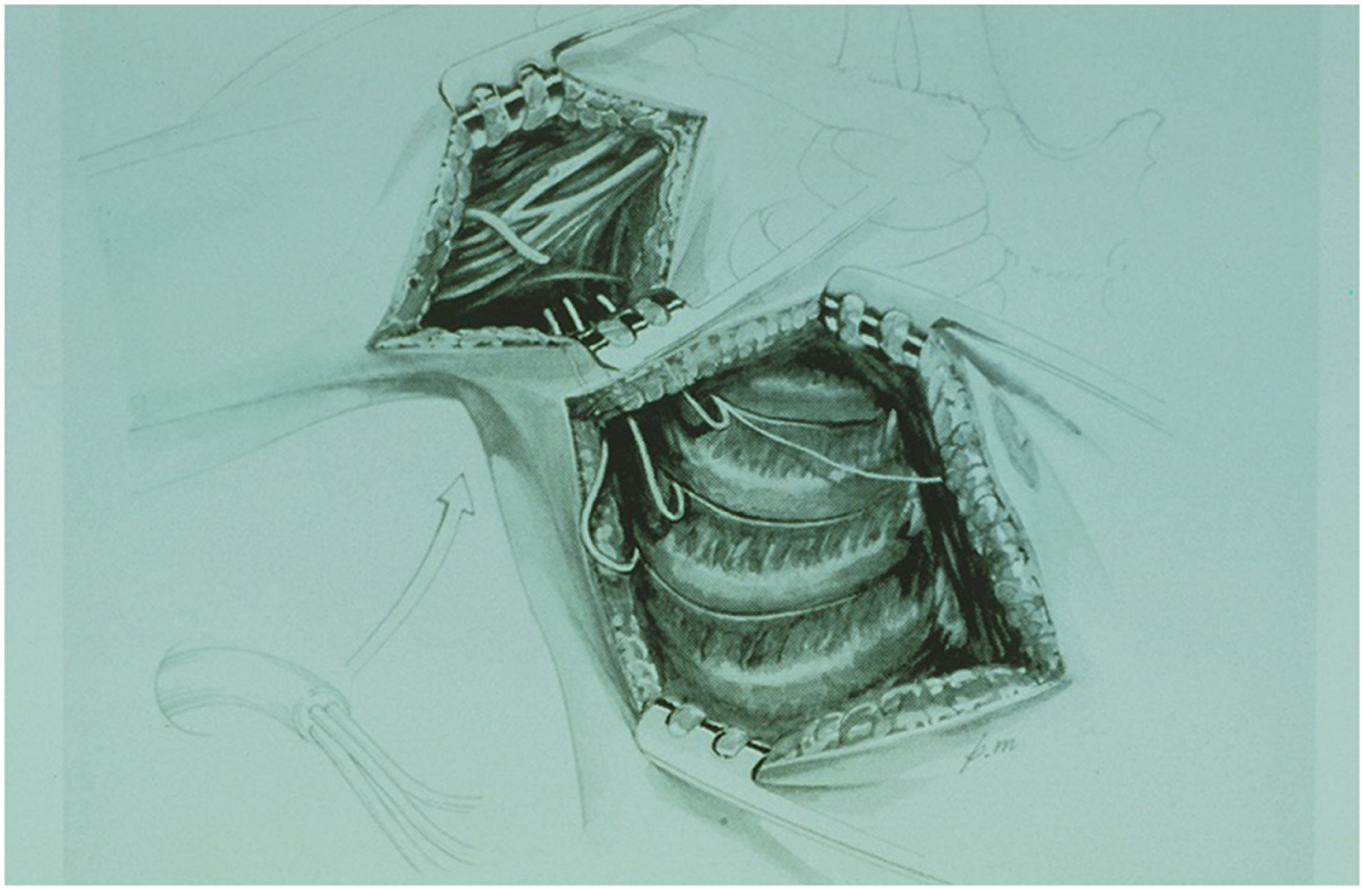
Submamry incisión for disecction intercostal nervs.

In total preganglionic lesions (C5-C6-C7-C8-T1) of the brachial plexus different extraplexual neurotizations are indicated. Mostly to reinervate total or partially the flexion of the elbow. Neurotization of the musculo cutaneous nerve by intercostal nerves (ICN) is a well known technique accepted with different outcomes [1,2]. The technique described by some authors is carried out by means of a large submammary skin incision (Figure 1) in order to obtain the third, fourth and fifth ICN. These ICN are drived to the musculo cutaneous nerve or it motor branches (biceps brachii muscle and brachialis muscle) and suture them directly or by grafts [3,4]. Based by previous authors [5,6] we describe the possibility of dissection and harvesting intercostal nerves by means of assisted video thoracoscopy (VATS-VIDEO ASSISTED THORACIC SURGERY) [5-8].

## Material and methods

The surgical method was planned in two groups, the first one in three cadavers (6 hemithorax) and the second group in three live pigs (mini-pigs breed of 30-40 Kgs. weight.

### Group 1 cadaver

**Figure 2:**
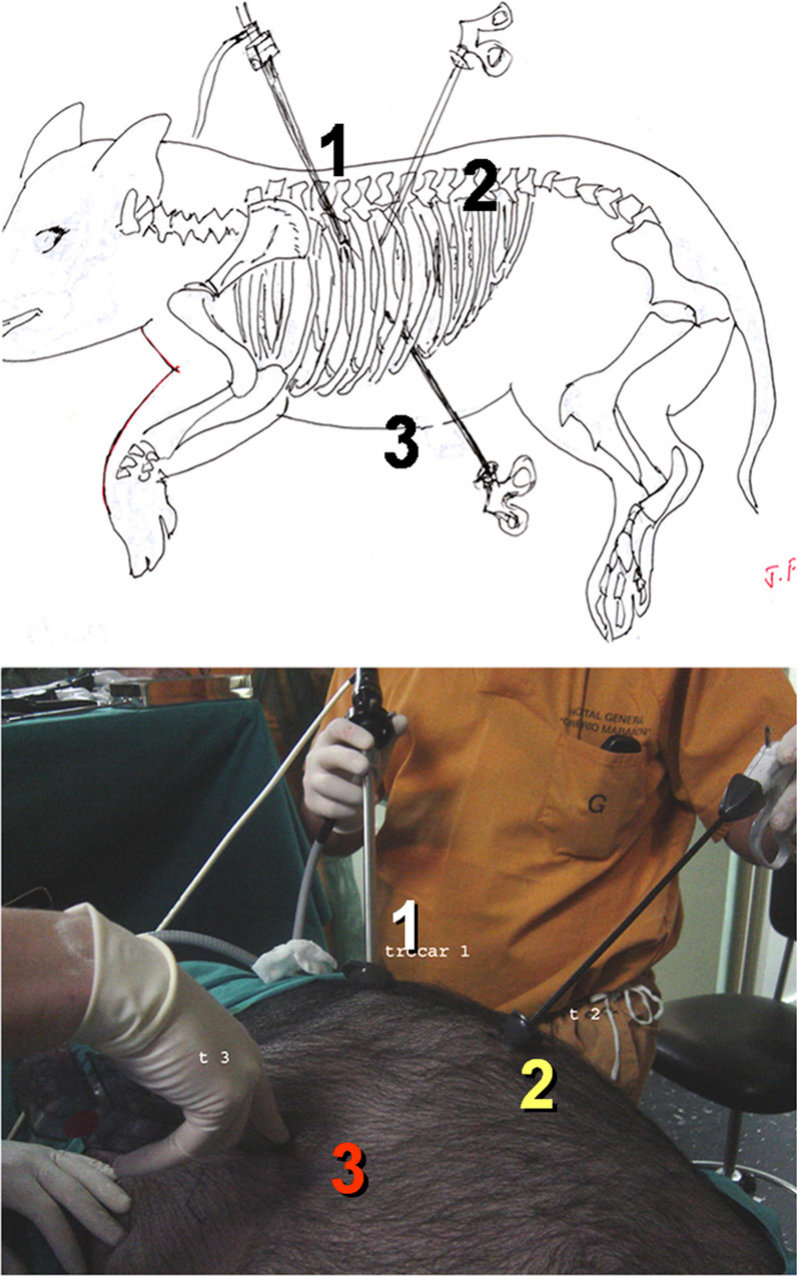
**Fress cadaveric.** Three portals 30º endoscopy in portal 1.

The trunk lays in lateral decubitus. Three portals of access are used. First trocar of diameter 10 mm at the level of the posterior axillary line: By this portal a fibroscopy with lens at 30° are introduced. Second trocar in the posterior scapular region. Third trocar at 3 or 4 cm parallel and anterior to the first access (Figure 2).

Prior of the dissection we localize the intercostal space between the third and fourth rib by an external puncture with a rachidian needle.

The internal mammary artery is taken as reference for the anterior limit of the dissection.

Incision the parietalis pleura, identification of the intercostal nerve and dissect it up to the axillary medial line. The same process is repeated for the next nerves until three or four of them are harvested.

Once freeded the nerves are externalised by a small incision at axillary level. At this point are used for the neurorraphy to the musculo cutaneous nerve.

Time employed: 30’ – 1 h for each hemithorax. (3 intercostal nerves).

### Group 2 (experimental animal)

Animals employed are male mini-pigs breed of 30 – 40 Kgs of weight. Well prepared operating theatre for experimental surgery and thoracoscopy tools [9-12].

**Figure 3:**
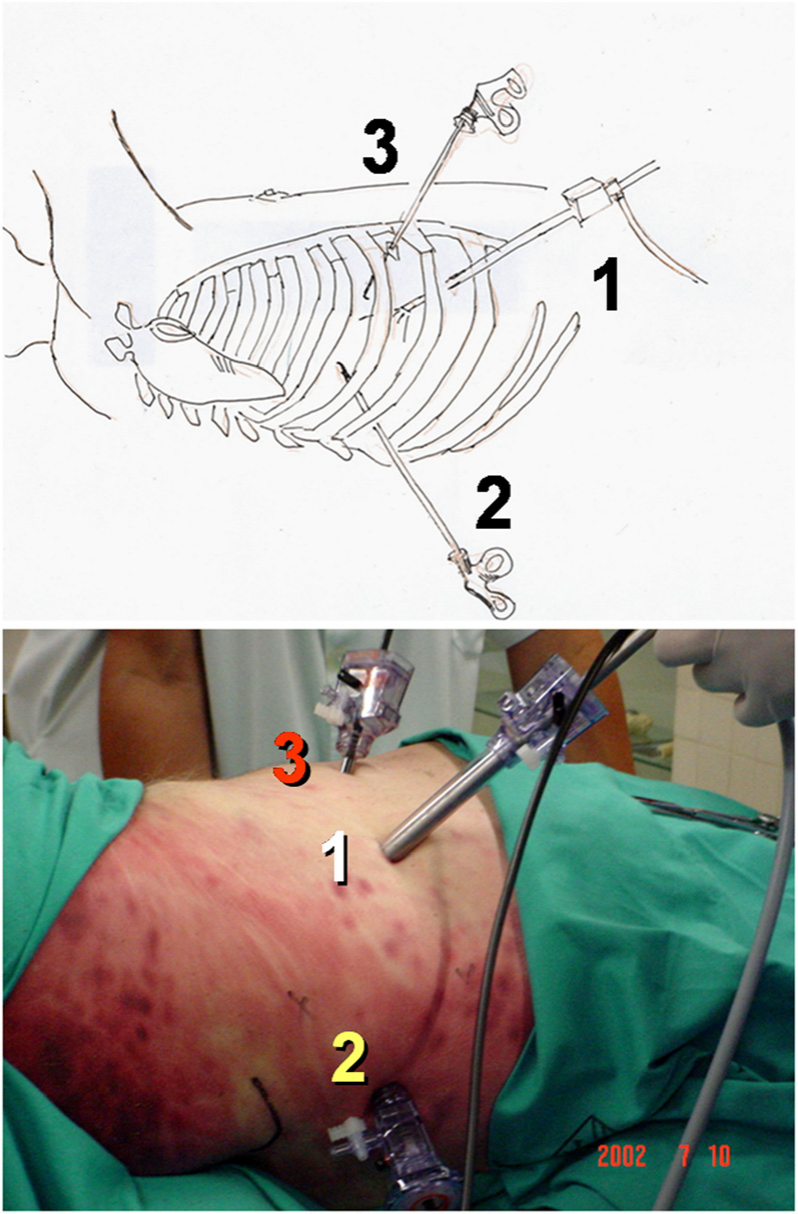
Mini-pig, endoscopy access.

The animal with selective endotracheal intubation lays in lateral decubitus. Three portals of access: a trocar of 10 mm for the thoracoscopy. Two of 5 mm for the surgical instruments (Figure 3) (hook and scissors with microcoagulation).

Dissection is realised at parietalis pleura after identification of the desired intercostal level.

Coagulation of intercostal vessels previous to the section of the nerve as anterior as the internal mammary artery allows it.

Same procedure is repeated until 3 or 4 nerves are harvested. Externalisation of them and glued by fibrine-glue so there are ready for nerve connection to the target. Normally the musculo cutaneous nerve or it motor branches. Animals were euthanized after the surgery to obtain information about the procedure.

Time employed 1 – 2 h in each hemithorax.

## Conclusions

In absence of previous studies concerning harvesting the intercostal nerves by VATS we demonstrated that there is a possibility to do that. In humans ICN III, IV, V are currently employed for neurotization of biceps muscle. ICN VI, VII for triceps. There are other options in total preganglionic lesions of the brachial plexus using parts (C7) of contralateral brachial plexus.

Solid clinical rules for harvesting intercostal nerves by means of VATS has still to be established.
